# A Registry Study of 240 Patients with X-Linked Agammaglobulinemia Living in the USA

**DOI:** 10.1007/s10875-023-01502-x

**Published:** 2023-05-23

**Authors:** Vivian Hernandez-Trujillo, Chuan Zhou, Christopher Scalchunes, Hans D. Ochs, Kathleen E. Sullivan, Charlotte Cunningham-Rundles, Ramsay L. Fuleihan, Francisco A. Bonilla, Aleksandra Petrovic, David J. Rawlings, M. Teresa de la Morena

**Affiliations:** 1 Division of Allergy and Immunology, Nicklaus Children’s Hospital, Miami, FL USA; 2 Allergy and Immunology Care Center of South Florida, Miami Lakes, FL USA; 3 Division of General Pediatrics, School of Medicine, Center for Child Health, University of Washington, Behavior, and Development, Seattle Children’s Research Institute, Seattle, WA 98145 USA; 4 Immune Deficiency Foundation. Immune Deficiency Foundation | (primaryimmune.org), Hanover, USA; 5 Division of Immunology, Department of Pediatrics, University of Washington, Seattle, WA 98101 USA; 6 Center for Immunity and Immunotherapies and the Program for Cell and Gene Therapy, Seattle Children’s Research Institute, Seattle, WA 98101 USA; 7 Department of Pediatrics, Children’s Hospital of Philadelphia, Philadelphia, USA; 8 Division of Allergy and Clinical Immunology, Departments of Medicine and Pediatrics, Icahn School of Medicine at Mount Sinai, New York, NY USA; 9 Division of Pediatric Allergy, Immunology and Rheumatology, Columbia University Medical Center, New York, NY USA; 10 Northeast Allergy Asthma and Immunology, Leominster, MA USA; 11 Department of Immunology, University of Washington, Seattle, WA 98101 USA

**Keywords:** X-linked agammaglobulinemia, XLA Registry

## Abstract

**Purpose:**

To understand the natural history and clinical outcomes for patients with X-linked agammaglobulinemia (XLA) in the United States utilizing the United States Immunodeficiency Network (USIDNET) patient registry.

**Methods:**

The USIDNET registry was queried for data from XLA patients collected from 1981 to 2019. Data fields included demographics, clinical features before and after diagnosis of XLA, family history, genetic mutation in Bruton’s tyrosine kinase (BTK), laboratory findings, treatment modalities, and mortality.

**Results:**

Data compiled through the USIDNET registry on 240 patients were analyzed. Patient year of birth ranged from 1945 to 2017. Living status was available for 178 patients; 158/178 (88.8%) were alive. Race was reported for 204 patients as follows: White, 148 (72.5%); Black/African American, 23 (11.2%); Hispanic, 20 (9.8%); Asian or Pacific Islander, 6 (2.9%), and other or more than one race, 7 (3.4%). The median age at last entry, age at disease onset, age at diagnosis, and length of time with XLA diagnosis was 15 [range (*r*) = 1–52 years], 0.8 [*r* = birth–22.3 years], 2 [*r* = birth–29 years], and 10 [*r* = 1–56 years] years respectively. One hundred and forty-one patients (58.7%) were < 18 years of age. Two hundred and twenty-one (92%) patients were receiving IgG replacement (IgGR), 58 (24%) were on prophylactic antibiotics, and 19 (7.9%) were on immunomodulatory drugs. Eighty-six (35.9%) patients had undergone surgical procedures, two had undergone hematopoietic cell transplantation, and two required liver transplantation. The respiratory tract was the most affected organ system (51.2% of patients) followed by gastrointestinal (40%), neurological (35.4%), and musculoskeletal (28.3%). Infections were common both before and after diagnosis, despite IgGR therapy. Bacteremia/sepsis and meningitis were reported more frequently before XLA diagnosis while encephalitis was more commonly reported after diagnosis. Twenty patients had died (11.2%). The median age of death was 21 years (range = 3–56.7 years). Neurologic condition was the most common underlying co-morbidity for those XLA patients who died.

**Conclusions:**

Current therapies for XLA patients reduce early mortality, but patients continue to experience complications that impact organ function. With improved life expectancy, more efforts will be required to improve post-diagnosis organ dysfunction and quality of life. Neurologic manifestations are an important co-morbidity associated with mortality and not yet clearly fully understood.

**Supplementary Information:**

The online version contains supplementary material available at 10.1007/s10875-023-01502-x.

## Introduction

X-linked agammaglobulinemia (XLA; OMIM #300,755) was first described in 1952 [1]. An inborn error of immunity, it is caused by pathogenic variants in the Bruton’s tyrosine kinase (*BTK*) gene, located on chromosome Xq21.3-Xq22 [2, 3]. The clinical phenotype is the consequence of a block in early B cell development, which results in absent/very low numbers of circulating B lymphocytes, low levels of serum immunoglobulins, and severely impaired antibody production. The disease prevalence is estimated between 1:100,000 and 1:200,000, but the true prevalence is not known, as newborn screening strategies have not been implemented. The clinical manifestations include recurrent infections with both bacterial and selective viral pathogens affecting the lungs, sinuses, ears, eyes, skin, joints, and brain. Patients can experience sepsis and bacteremia with encapsulated pathogens and *Pseudomonas* spp., and gastrointestinal infectious diseases are frequently encountered [4–6]. Series of patients with XLA from different countries confirmed similar infectious patterns independent of their geographic location [5–11]. Meningoencephalitis caused by enteroviruses is reported in industrialized countries, while vaccine-associated paralytic polio remains a threat in those countries where live-attenuated polio vaccine remains the standard of care [7]. Antimicrobial therapies and lifelong immunoglobulin replacement (IgGR), in countries where these are easily available, have allowed patients to survive into adulthood. As patients age, non-infectious complications such as inflammatory bowel disease, autoimmunity, and malignancy are being recognized, many times without effective therapies. Overall mortality varies depending on geographic location, but survival beyond 20 years is reported to be lowest in Asia and Africa [7, 8, 12].

The USIDNET registry database is a compilation of information from medical centers around the USA and Canada. USIDNET, a program of the Immune Deficiency Foundation (IDF), is supported by a cooperative agreement, U24AI86837, from the National Institute of Allergy and Infectious Diseases (NIAID). The current on-line patient-consented USIDNET registry compiles de-identified data and is available for research purposes after a query request is submitted to the USIDNET Steering Committee. The last similar report on XLA from this registry was published 15 years ago and included 201 patients. Within this cohort, the age of diagnosis without a family history was 5.4 years, infections were recognized as the most common presentation, and 8.5% (17/201) had died [6]. The purpose of this study is to understand if clinical outcomes and treatment of XLA among US-based patients have significantly changed in the past 15 years.

## Methods

Data was extracted from de-identified data provided by the USIDNET registry. A total of 49 medical centers and fifty medical providers in the USA contributed data on patients with XLA. We confirmed that the data provided by USIDNET for this analysis did not include the original legacy patient datasets utilized and published by Winkelstein et al. in 2006 [6]. Data fields included year of birth, age at disease onset, age at diagnosis, living status, age of death, length of time with diagnosis of XLA, age when treatment was started, race/ethnicity, family history, number of family members with primary immunodeficiency, and gene mutation. This registry also collects patient treatments including use of IgGR and route of administration, antibiotic prophylaxis, immunomodulatory drugs, surgeries, and whether patients had undergone either hematopoietic cell transplantation (HCT) or solid organ transplant.

Patient data entered by their medical provider included infections and conditions involving different organ systems (Supplemental Table 1). Affected organ systems included the central nervous system, respiratory, cardiovascular, gastrointestinal, renal, musculoskeletal, hematologic, endocrine, and psychiatric aspects. Types, locations of infections, and pathogens identified were reviewed. Infections were recorded based on location in the body. Sinopulmonary infections included pneumonia, sinusitis, and otitis media. Skin infections, conjunctivitis, abscess, opportunistic infections, sepsis, meningitis, bacterial arthritis, encephalitis, osteomyelitis, and bacteremia were compiled.

### Statistical Analysis

We conducted a detailed descriptive analysis of the USIDNET XLA cohort. We wanted to characterize the types of infections, co-morbid conditions, and sub-conditions within different organ/body systems. We reported means and standard deviations on continuous variables for highly skewed continuous variables, and we reported median and inter-quartile ranges. For categorical variables, we reported counts and proportions. We further examined the effects of IgG dose on survival, infection type, and complications using multivariable linear and generalized linear regression models. All analyses were conducted within R statistical software [13].

## Results

### Patient Cohort

Data on 240 patients with XLA living in the USA were entered between the years 1981 and 2019 (Table 1). While the registry was created in 1999, data from prior visits based on medical records were included which dated back as early as 1981. Patients were born between 1945 and 2017 (median, 1991; range, 1945–2017). One hundred and forty-one patients (58.7%) were < 18 years of age.

**Table 1:** Demographics, patient characteristics, and treatment modalities for the USIDNET XLA patient cohort

Demographics	
Year of data entry	1981–2019
Year of birth (median; range)	1945–2017 (1991; 1945–2017)
Race (*N* = 204)	
White/Caucasian	148 (72.5%)
Black/African American	23 (11.2%)
Hispanic	20 (9.8%)
Asian or Pacific Islander	6 (2.9%)
Other or more than one race	7 (3.4%)
Living status (*N* = 178)	
Alive	158 (88.8%)
Deceased	20 (11.25)
Patient characteristics	
Family history (*N* = 180)	112 (62%)
Reported mutation in BTK	229 (95%)
Age at last entry: years (yrs); median (range)	15 (1–52)
Age at disease onset (*N* = 123): yrs; median (range)	0.8 (birth–22.3)
Age at diagnosis (*N* = 191): yrs; median (range)	2 (birth–29)
Length of time with XLA diagnosis: yrs; median (range)	10 (1–56)
Body mass index (mean, SD)	25.6 (6.61)
Treatment modalities	
IgG therapy (%)	221 (92)
Prophylactic antibiotics (%)	58 (24)
Immunomodulators (%)	19 (7.9)
Surgeries (%)	86 (35.9)
Hematopoietic cell transplantation (%)	2 (0.8)
Liver transplantation (%)	2 (0.8)

Race was indicated in 204 patients as follows: White, 148 (72.5%); Black/African American, 23 (11.2%); Hispanic, 20 (9.8%); Asian or Pacific Islander, 6 (2.9%); and other or more than one race, 7 (3.4%).

Living status was reported for 178, of whom 158 (88.8%) were alive at the time of this analysis. One hundred and twelve (62%) patients reported a family history of immune deficiency among 180 with documented family history. Of those patients with a reported family history, a primary immune deficiency (PID) was reported by one other family member in 17.5%, 2 other family members in 10.4%, and more than 2 other family members in 7.9% of patients. *BTK* was the reported affected gene in 229 (95%) of the patients. However, the *BTK pathogenic variant* was only documented in 25 patients (Supplemental Table 12).

The median age at last entry was 15 years (range, 1–56). The median age of disease onset was 0.8 years (range, birth–22), with a median age at diagnosis of 2 years (range, birth–29), and the median length of time with diagnosis of XLA was 10 years (range, 1–56). The mean BMI for adult patients in this cohort was 25.6 (SD = 6.61) while in the USA, the average adult male has a BMI of 26.6 (https://www.cdc.gov/nchs/data/nhanes/databriefs/adultweight.pdf).

The racial/ethnic breakdown reported by this USIDNET cohort (White, 72.5%) differs from what is currently being reported by the US Census Bureau (https://www.census.gov/quickfacts/fact/table/US/PST045221). Therefore, a subset analysis was performed to look at differences between White and non-White. For the limited data that was available, no difference was identified for age of death, age of disease onset, age at diagnosis, age at start of IgGR, family history, and survival (Supplemental Table 14).

Concerning treatment modalities, 221 (92%) patients were reported as receiving IgGR. For 138/221 patients, the mode of delivery was reported as intramuscular (IM) in 1 patient, intravenous (IV) in 92 patients, and subcutaneous (SC) in 45 patients. We were unable to determine the number of patients who may have been on more than one mode of IgG delivery (i.e., IV switched to SC or SC switched to IV and IM), due to the limitations of the dataset. Fifty-eight patients were reported as receiving antibiotic prophylaxis as shown in Supplemental Table 13. Two patients were reported as having received palivizumab. The IgG status was available for 49/58 patients as follows: 30 (61%) were on IVIG and 19 (39%) were on SCIG. The indication for prophylaxis and length of prophylaxis could not be extracted from the dataset. Nineteen patients (7.9%) were on treatment with immunomodulatory drugs (Supplemental Table 11). Eighty-six (35.9%) patients had undergone a total of 179 surgical procedures (data not shown), two had undergone hematopoietic cell transplantation, and one patient was reported as living with an unknown status for the other. Liver transplantation had been reported for 2 patients, both of whom were alive (Table 1).

### Infections

**Fig. 1:**
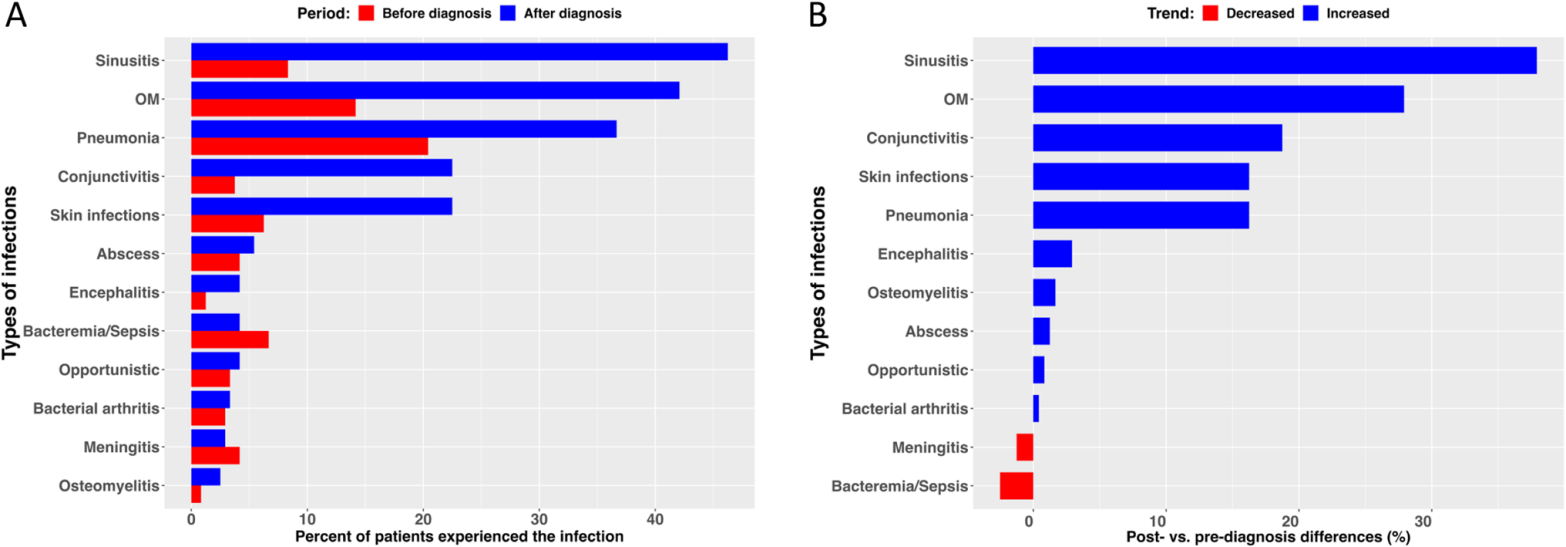
**A** Prevalence of different types of infections before and after diagnosis, sorted by post-diagnosis prevalence. **B** Post- vs. pre-differences in prevalence for each type of infection. For example, bacteremia/sepsis and meningitis infections had the largest reduction after diagnosis, as shown at the bottom of the graph. OM = otitis media

In this cohort, 222 patients (92.5%) reported infections, with 1054 total infections reported in the Registry (Supplemental Table 2). Most patients (56.2%) reported pneumonia, while similar numbers reported sinusitis (54.6%) and otitis media (54.2%). Common infections included skin infections (29.2%), conjunctivitis (25%), and abscess (10%). Ten percent of patients reported bacteremia (7.5%) or sepsis (2.5%). Opportunistic infections were recorded in 8.3%, but details of these infections could not be clearly ascertained. Seven and a half percent of patients had meningitis, while bacterial arthritis and encephalitis were noted in 5.8% each. When comparing infections prior to diagnosis versus those after diagnosis, pneumonia was more commonly noted before diagnosis, followed by otitis media and sinusitis. However, after the diagnosis of XLA was established and despite IgGR, patients continue to experience episodes of pneumonia, but sinus and ear infections were more frequent (Fig. 1A). When evaluating post- vs pre-diagnosis differences in prevalence for each type of infection, only meningitis and bacteremia/sepsis had reduction after the diagnosis of XLA was made (Fig. 1B).

### Medical Conditions and Organ involvement

Medical conditions other than infections were reported in 193 patients (80.4%), with 1243 total conditions reported (supplemental Table 1). After diagnosis, 178 patients reported any medical condition: 89 patients (50%) reported four or more conditions, 40 (22%) reported 1 condition, 28 (15.7%) reported two conditions, and 21 (11.8%) reported 3 conditions (data not shown).

**Fig. 2:**
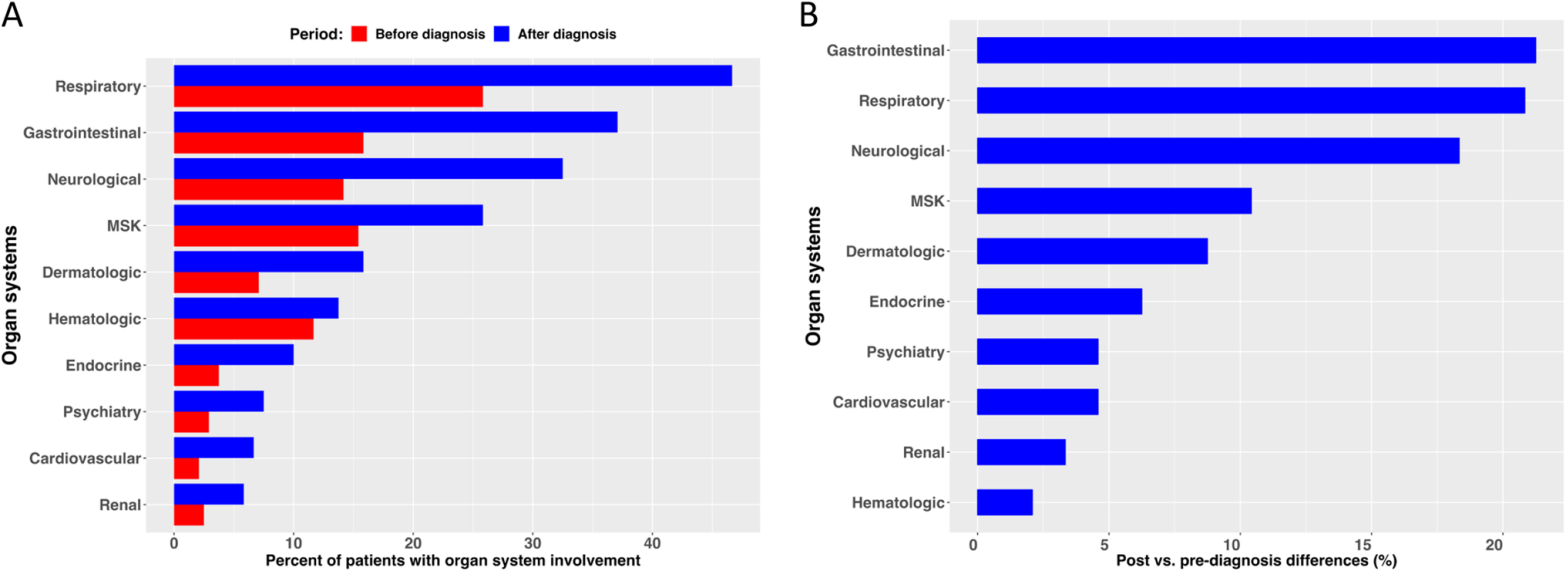
**A** The prevalence of body system involvement before and after XLA diagnosis. **B** Post- vs. pre-differences. The three body systems with the largest increases after diagnosis are gastrointestinal, respiratory, and neurological. MSK = musculoskeletal

XLA patients were reported as having different organ systems involved (Table 2). The highest number of patients (51.3%) experienced respiratory illnesses, followed by gastrointestinal (40.0%), neurological (35.4%), and musculoskeletal (28.3%) problems (Table 2). Other conditions reported included hematologic (19.2%), dermatologic (18.3%), endocrine (10.8%), cardiovascular (7.9%), psychiatric (7.5%), and renal involvement (7.1%). (Supplemental Tables 3–10). When comparing organ system involvement before and after diagnosis of XLA, respiratory and gastrointestinal manifestations continued to be reported, but both neurological and musculoskeletal complications appeared to be more frequently noted after the diagnosis of XLA was made and despite therapies (Fig. 2A). When post- vs. pre-diagnosis differences were investigated, the three body systems with the largest increases after diagnosis were gastrointestinal, respiratory, and neurological (Fig. 2B). Among patients with respiratory disease, lower respiratory disease, asthma, bronchitis, and COPD were most frequently reported. In this cohort, twenty-two patients reported bronchiectasis (9%) (Supplemental Table 3). Among gastrointestinal manifestations, diarrhea, abdominal pain, and GERD were frequent. Inflammatory bowel disease, including Crohn’s disease, colitis, and ulcerative colitis, was seen in 14 patients (14/240; 5.8%; Supplemental Table 4). Among patients with neurologic complications, more than half had reported cognitive disabilities, speech delay, mental retardation, and difficulties with educational academic activities. Thirteen of these patients were affected by seizures/epilepsy. Among this cohort, 5 were reported as having had paralytic polio, all born prior to 1991 (Supplemental Table 5). Most patients with musculoskeletal symptoms described arthritis, arthralgias, or generalized aches and pains. Five patients are reported as having dermatomyositis. (Supplemental Table 6). Interestingly, of the five patients reported as having dermatomyositis, two had died and had experienced encephalitis and encephalopathy respectively; another patient had reported “enterovirus” among the infectious history. Sinusitis and no infections were documented for the other two patients. For those patients with hematologic organ involvement (46/240), all but one had some form of cytopenia; in 6 patients, more than one cytopenia was reported, and 1 patient had a B cell lymphoma (Supplemental Table 7). The most common endocrine abnormality was short stature with or without growth hormone disorder (Supplemental Table 8). Psychiatric manifestations included attention deficit disorder with hyperactivity followed by depression, behavioral problems, and anxiety (Supplemental Table 9). Almost 8% of patients reported a cardiac condition (Supplemental Table 10). Less commonly affected organs were the kidneys and liver (data not shown).

**Table 2:** Organ/system involvement in USIDNET XLA patients

	Patient cohort *N* = 240 (%)
Respiratory	123 (51.25)
Gastrointestinal	96 (40.00)
Neurological	85 (35.42)
Musculoskeletal	68 (28.33)
Hematologic	46 (19.17)
Dermatologic	44 (18.33)
Endocrine	26 (10.83)
Cardiovascular	19 (7.92)
Psychiatry	18 (7.50)
Renal	17 (7.08)

Symptoms of allergic disease such as chronic rhinitis, eczema/atopic dermatitis, and urticaria were reported in 28% of patients. Food allergies were reported in 7 patients and 44 patients reported drug allergies. Anaphylaxis was reported in 4 patients (2 to shellfish, 1 to bee venom, and 1 to sulfamethoxazole-trimethoprim) (data not shown).

Rare conditions included 2 patients with bronchiolitis obliterans with organizing pneumonia (BOOP), 4 with appendicitis, and 1 patient with neurofibromatosis. Among this cohort, 3 (1.25%) patients reported malignancy (B cell lymphoma, angiosarcoma, and craniopharyngioma).

### Treatment with Anti-microbials, Immunoglobulin Therapy, Immunomodulating Medications, Surgery, and Transplantation

In this cohort, 58 patients (24%) received antibiotic prophylaxis, and 44 of them were reported as being on continuous treatment. Regression analysis showed that patients on continuous prophylactic antibiotics were more likely to have an increased number of conditions (rate ratio (RR), 1.17; 95% CI, [1.10, 1.24]; *p* < 0.001). We did not detect a significant correlation between infection and continuous prophylactic antibiotic use (OR, 1.2; 95% CI, [005, 11.0], *p* = 0.9; data not shown). Most (92%) patients reported receiving gamma globulin treatment at some point.

**Fig. 3:**
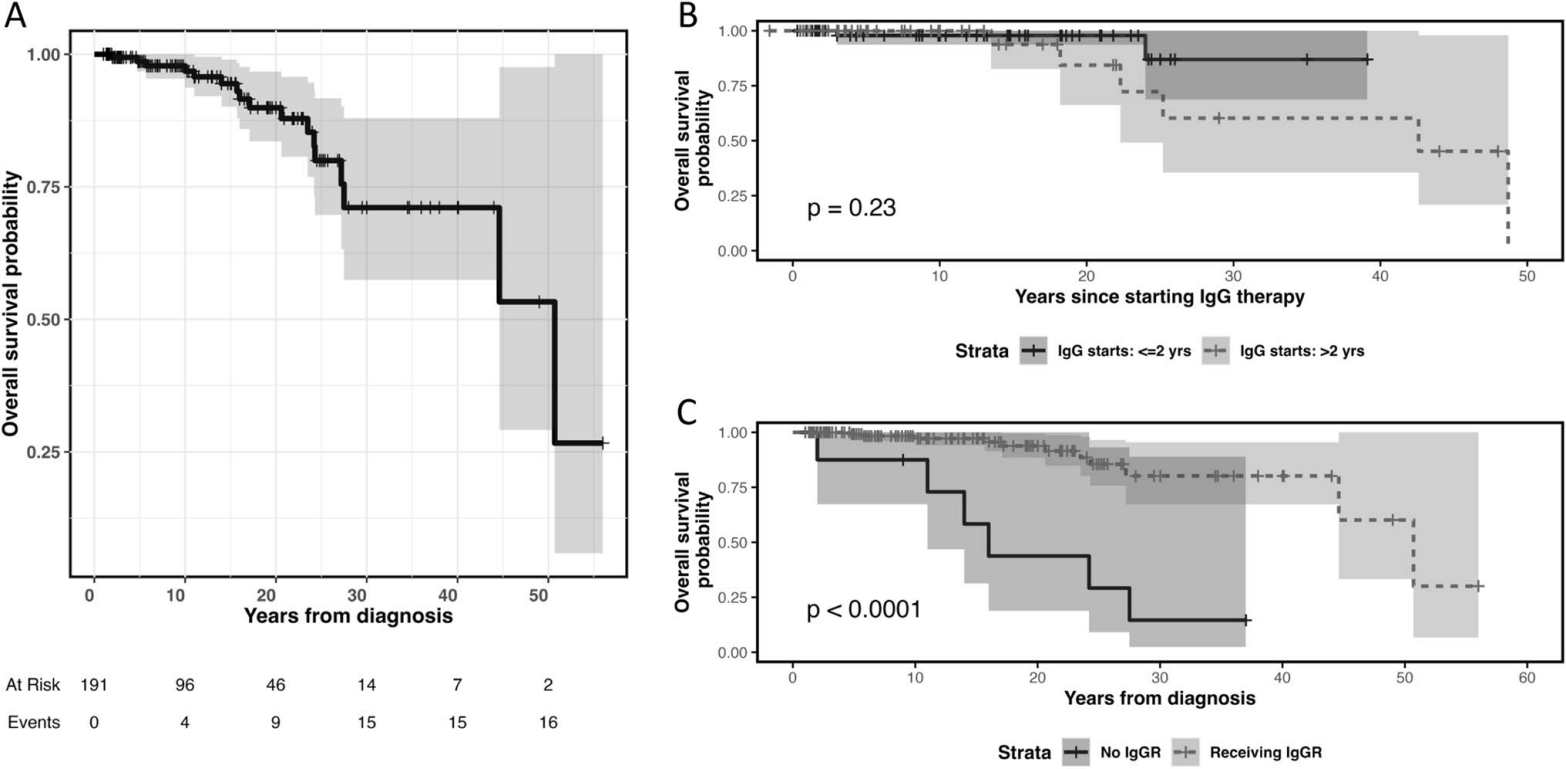
KM survival analysis of US XLA patients. **A** Overall survival (*N* = 191); **B** probability of survival for 117 XLA patients comparing IgG therapy starting before (*N* = 62) or after (*N* = 55) 2 years of age; **C** probability of survival for 191 XLA patients comparing patients with (*N* = 180) or without (*N* = 11) IgGR therapy

Headaches were the most common IgG therapy reactions reported in 220 patients. Serious reactions included 2 patients with anaphylaxis, while thromboembolism, renal dysfunction, and aseptic meningitis were reported in 1 patient for each condition. In this cohort of patients, after controlling for age at the time of first IgG treatment, no significant association between IgG dose (mg/kg) and IgG level (mg/dl) and years of survival was observed since initial treatment (data not shown). IgG replacement therapy had a significant positive impact on survival (years lived since diagnosis). Patients in whom a response to “ever receiving IgGR” after diagnosis was either “no” or “not documented” (*N* = 11) had a median survival of 16 years, compared to 51 years for those who ever received IgG therapy (*N* = 180). Cox regression showed that IgG therapy was associated with 89% reduction in risk of death (hazard ratio = 0.11, *p* < 0.001; Fig. 3C).

The use of immunomodulators was reported in 8% of patients. Of note, three of the patients were on more than one immunomodulator (Supplemental Table 11).

Eighty-six (36%) patients underwent a total of 179 surgical procedures. Of these, 12% underwent either tonsillectomy or adenoidectomy, two patients had both, and all except one had a reported *BTK* as the gene affected. Other patients reporting to have had surgery underwent placement of myringotomy tubes (30%), Port-a-Cath (9%), and appendectomy in 12.6% patients (data not shown).

Four patients had received an organ transplant: two underwent HCT and two had a liver transplant. One of the two patients treated with HCT reported it as treatment for the primary immunodeficiency and was alive. The living status of the other patient who had undergone HCT was not reported. One liver transplant patient reported indication for end-stage cirrhosis and the other for portal hypertension; both were reported as living.

### Survival

Of 178 patients with reported living status, 158 (88.8%) were living and 20 (11.2%) were deceased. For 191 patients in whom sufficient data was available (age at diagnosis, living/death status, and most recent office visit date), Kaplan–Meier survival analysis showed that 75% of patients had survived to 25 years beyond diagnosis (Fig. 3A). There was no significant difference in survival between those who started IgG before or after 2 years of age, which corresponds to the median age at diagnosis among this cohort (Fig. 3B).

The reported median age of death was 21 years (range, 3–56.7 years). On average, these patients were diagnosed at a median age of 2.7 years of age (range, 0.8–8.7 years) and had lived to a median age of 17.1 years (range, 2–50.7) since diagnosis. In 14 of these 20 (70%) families, a history of XLA was known, whereas IgGR status was not known for 6/20 deceased patients. The most common underlying co-morbid conditions in these deceased patients were neurologic (75%), followed by respiratory (70%) (Table 3). Causes of death were primarily infections. Most of the deaths reported occurred before 1993, the year the *BTK* gene was discovered. Since the year 2000, only one death is reported among this USIDNET cohort.

**Table 3:** Underlying co-morbid conditions in deceased XLA patients

Co-morbid conditions	Total number of patients *N* = 20 (%)
Neurological	15 (75)
Respiratory	14 (70)
Gastrointestinal/liver	13 (65)
Musculoskeletal	13 (65)
Cardiovascular	7 (35)
Dermatologic	7 (35)
Hematologic	4 (20)
Psychiatry	2 (10)
Endocrine	2 (10)
Renal	1 (5)

The Lansky and Karnofsky scales of daily living were collected for 46 and 30 patients respectively. The mean Karnofsky score was 90.6 ± 10.99 and the mean Lansky score was 92.38 ± 21.3.

## Discussion

We sought to understand the health impacts experienced by XLA patients living in the USA, utilizing the USIDNET registry. The racial/ethnic breakdown of patients included in this dataset is predominantly White (72.5%) and is not a complete representation of the current US population based on the most recent US census Bureau that shows White, 59.3%; Black/African, 13.6%; Hispanic/Latino, 18.9%; Asian, 6.1%; American Indian or Alaska Native, 1.3%; Native Hawaiian or Pacific Islander, 0.3%; and two or more races, 2.9% (https://www.census.gov/quickfacts/fact/table/US/PST045221). As new registries are developed, efforts should be made to ensure inclusion of all ethnic/racial backgrounds, along with gender and socioeconomic and insurance status to allow for a better understanding of how health disparities impact morbidity and mortality.

Compared to the previous analysis of US patients performed utilizing the USIDNET registry in 2006, the current cohort of XLA patients was diagnosed at a younger age (median age, 2 years), had a higher reported family history (62%), and in 95% of cases, a molecular diagnosis had been established as compared to 59% of patients from an earlier cohort [6].

In the absence of newborn screening for XLA, a delay in diagnosis can impact long-term morbidity [14]. Improvement in early diagnosis is notable among this group of XLA patients residing in the USA, when compared to previous US reports and those from other regions [6, 7, 9, 14]. However, until newborn screening (NBS) programs incorporate analytes to detect congenital B cell deficiencies, earlier diagnosis will likely not change, and true prevalence will remain unknown [15].

Infections remain the critical contributor to morbidity and organ dysfunction in patients with XLA [6, 7, 9, 16]. After almost 60 years of IgG replacement therapy, there is no doubt to its critical role for the survival of XLA patients (Fig. 3C). Yet, the most striking observation among this cohort is the frequent episodes of infections and organ system involvement that patients with XLA continue to experience, despite IgG replacement and supportive therapies. This is not a novel finding and corroborates similar observations reported by a carefully designed study by the Italian Primary Immunodeficiency Network (IPINET) [9]. In addition, 50% of patients from this USIDNET cohort were reported as having more than three medical conditions after diagnosis, an observation that has been implicated as impacting quality of life [17]. Specifically, before or at diagnosis, 20.4% of patients experienced pneumonia. However, after diagnosis and IgGR, pneumonia continued to occur in almost 40% of patients, but sinusitis (46.2%) and otitis media (42.1%) became more frequent, followed by skin infections (22.5%) and conjunctivitis (22.5%), both having increased dramatically (Fig. 1A and supplemental Table 2). Twenty-two patients (9.1%) were reported as having bronchiectasis. This dataset did not allow us to establish whether bronchiectatic changes were present at diagnosis or during a patient’s lifetime. A recent report analyzing the same registry of patients found that the earlier the patients were diagnosed, the less likely they were to develop lower respiratory tract infections [16]. This is important because once bronchiectatic changes have occurred, disease progression is difficult to control and leads to chronic lung disease (CLD), a known predictor of morbidity and mortality [5, 7, 9]. However, recent reports indicate the development of CLD despite IgG replacement [9]. Enterovirus is a well-known cause of infections, particularly encephalitis, in patients with XLA [14, 18]. Extraordinarily, encephalitis was four times more likely to occur after diagnosis in this cohort and higher than previously reported by the Italian cohort [9]. The reason for this is unknown but highlights caution when comparing findings from studies with different study design. In addition, this may also underline the importance of BTK in the immune response to certain viruses [19, 20]. The dramatic increase of neurologic system involvement after diagnosis as the third most commonly reported system affected merits additional investigations (Fig. 2B). Fortunately, there was an almost 50% reduction in cases of bacteremia/sepsis/meningitis after the diagnosis of XLA was made. However, these serious life-threatening infections were still reported (Fig. 1A, B; Supplemental Table 2).

Inflammatory bowel disease, Crohn’s disease, colitis, and ulcerative colitis were collectively seen in 14 patients (14/240; 5.8%; Supplemental table 4). A previous US cohort from 2006 reported diarrhea in over 50% of patients, but inflammatory colitis was not called out [6]. A more recent update among US patients entered in the USIDNET registry identified a higher prevalence of IBD or enteritis [21]. The description of the different gastrointestinal manifestations reported makes it difficult to compare to other series, but in general, US patients appear to report less inflammatory enteritis [9–11] [21]. Similarly, autoimmune manifestations such as arthritis, cytopenias, and endocrinopathies were identified, many of which have been previously recognized [22]. XLA patients with dermatomyositis merit further investigation to understand if viruses, like enterovirus, are potentially important triggers.

Among patients in this cohort, an unexpected number of reports of appendicitis, above that expected in the general population of 1 in 1000 patients, were observed. Similarly, despite the dogma that XLA patients have small/undetectable tonsils, it was unusual to identify 12% of XLA patients undergoing either tonsillectomy or adenoidectomy. There was insufficient information to evaluate whether these patients represented a “leaky” XLA phenotype.

Interestingly, several patients experienced features of atopy and food and drug allergies, an aspect to further examine. Many conditions involving the central nervous system were identified in this cohort with patients reported as having cognitive delays, seizures, mental retardation, and learning difficulties. There is no clear understanding of whether these observations are the result of infectious injury and will require further inquiry. Similarly, mental health concerns such as depression, anxiety, drug abuse, and suicidal ideation were noted among this US cohort, but has received little attention (Supplemental Table 5). Studies are needed to address mental health concerns in PID in general.

Mortality in the past 20 years appears improved among this US cohort, with only one death since 2000. This is in contrast to large multicenter international studies [7] [11]. However, this observation may be limited by selection bias as those patients entered are primarily followed by subspecialists very familiar with their condition.

Few patients in the USIDNET registry underwent transplantation, with only two patients having had bone marrow transplantation and two patients liver transplantation. Reports of patients undergoing stem cell transplantation for XLA have been few [23, 24]. None of the patients in this cohort had undergone lung transplantation [25].

This study has several limitations. First, the racial/ethnic breakdown of patients included in this dataset is predominantly White (72.5%) and is not a complete representation of the current US population. A subset analysis did not identify a difference between White and non-White with respect to age of death, age of disease onset, age at diagnosis, age at start of IgGR, family history, and survival (Supplemental Table 14). However, the data is limited and further studies are needed to investigate whether differences may exist. Second, health disparities were not adequately captured and we were unable to address how ethnic/racial backgrounds, along with socioeconomic status and insurance coverage, impacted access to care and subsequently morbidity and mortality. As new registries are developed, efforts should be made to include information regarding social determinants of health to allow better understanding of how health disparities are impacting patient outcomes. Third, our study may suffer from selection bias as those patients included represent patients followed primarily by clinical immunologists from large academic referral centers or large private practice groups with experience caring for XLA patients. Fourth, while more XLA patients are reported as having a *BTK* defect as compared to 15 years ago, it was disappointing to see that the exact pathogenic variant was not available for most and its correlation with clinical outcomes could not be analyzed. Fifth, the dataset lacks continuous long-term follow-up data and the number of patients with complete information on IgG dosing/route of administration/trough levels, and details on specific infections, was small making it difficult to perform statistical analysis with meaningful clinical correlations due to lack of power and/or targeted study design.

There is no doubt that current therapies in XLA patients reduce early mortality, but patients continue to experience complications that impact both organ function and health-related quality of life (Fig. 2B) [9, 26]. Sinopulmonary infections and hospitalizations remain part of the day-to-day life for these patients. Among this cohort of patients, no significant association was detected for IgG dose and/or IgG level and survival, but this is likely due to limited data among those who died.

As patients with XLA live into adulthood, there is a need to establish standards of practice to monitor progression of disease and end organ damage. The cumulative risk of chronic lung disease in XLA has been reported to reach 47% at 50 years of age [9]. Therefore, how should lung function of XLA patients be monitored? Should all patients have periodic CT imaging? What about pulmonary function studies? When should this start and how frequently? Given the gastrointestinal and neurologic manifestations reported, should efforts be directed at identifying biomarkers for these complications? Newborn screening strategies are currently being considered [27–29]. Earlier diagnosis could allow for early institution of therapies. However, it is clear from this and the longitudinal study of Italian patients that IgG replacement during the lifetime of these patients is not sufficient to control morbidity and mortality [9, 16]. Alternatively, gene therapy strategies are in the horizon. B cell development and function were restored in a murine model of XLA using UCOE-BTK promoter–based lentiviral gene therapy with a recent publication describing its safety and efficacy, and sustained correction of B cell function in these murine models [30–32].

## Conclusions

In this large-cohort study of patients with XLA in the United States Immunodeficiency Network registry, patients have frequent co-morbid conditions and infections, despite the use of different treatment modalities. Patients with XLA need alternative early diagnosis and novel treatment options to address the underlying disease and aid in the treatment and prevention of co-morbid conditions that complicate the health and impact the quality of life of these individuals.

## Supplementary Information

Below is the link to the electronic supplementary material.

Supplementary file1 (DOCX 40 KB)

## Data Availability

The database used and analyzed for the current study is available from the corresponding author upon reasonable request.

## Code Availability

The statistical analyses performed, using the R statistical software, are available from the corresponding author and statistician upon reasonable request.
