# Supplementary material for: A Registry Study of 240 Patients with X-Linked Agammaglobulinemia Living in the USA
Source: J Clin Immunol. 2023 May 23;43(6):1468–77. doi: 10.1007/s10875-023-01502-x (PMC10354121; doi:10.1007/s10875-023-01502-x)
Supplement: Supplementary file 1 — Supplementary file1 (DOCX 40 KB) [file 10875_2023_1502_MOESM1_ESM.docx]

Supplemental Table 1. Conditions by Organ/Body system

| **Organ/System** | **Specific Conditions** |
| --- | --- |
| Respiratory |  |
| Upper Respiratory |  |
|  | Allergic rhinitis |
|  | Croup |
|  | Nasal congestion |
|  | Rhinorrhea |
| Lower respiratory | Asthma |
|  | BOOP |
|  | Bronchiectasis |
|  | Bronchiolitis |
|  | Bronchitis |
|  | Clubbing |
|  | COPD |
|  | Cough |
|  | Diffuse Alveolar Hemorrhage |
|  | Dyspnea |
|  |  |
| Gastrointestinal | Abdominal pain |
|  | Appendicitis |
|  | Biliary dyskinesia |
|  | Cirrhosis |
|  | Cholecystitis |
|  | Colitis- IBD/Crohn’s/Ulcerative colitis |
|  | Constipation |
|  | Diarrhea |
|  | Diverticulitis |
|  | Esophagitis |
|  | Failure to gain weight |
|  | Failure to thrive |
|  | GERD |
|  | Gastroenteritis |
|  | GI fistula |
|  | Gilbert syndrome |
|  | GI obstruction |
|  | Hemangioma of liver |
|  | Hematochezia |
|  | Hepatomegaly |
|  | Hyperbilirubinemia |
|  | Obesity |
|  | Portal hypertension |
|  | Splenomegaly |
|  | Steatosis of liver |
| Neurologic | Aphasia, |
|  | Apraxia |
|  | Asthenia |
|  | Cerebellar ataxia |
|  | Cognitive disability |
|  | Complex regional pain syndrome |
|  | Deafness-dystonia-optic neuropathy |
|  | Decreased taste |
|  | Degenerative brain disease |
|  | Dystonia |
|  | Educational problems |
|  | Headache |
|  | Iritis |
|  | Milestone delay |
|  | Neurofibromatosis |
|  | Polio |
|  | Seizures/epilepsy |
|  | Speech delay |
| Musculoskeletal | Arthralgia |
|  | Arthritis |
|  | Dermatomyositis |
|  | Generalized aches and pain/ malaise |
|  | Scoliosis |
| Hematologic | B cell lymphoma |
|  | Cytopenia- neutropenia, anemia, thrombocytopenia |
|  | HLH |
|  | Petechiae |
| Endocrine | Diabetes mellitus |
|  | Short stature/ growth hormone disorder |
|  | Thyroid disease |
| Psychiatric | ADHD |
|  | Anxiety |
|  | Autism |
|  | Behavior problems |
|  | Depression |
|  | Drug abuse |
|  | Socialized behavior disorder |
|  | Suicidal ideation |
| Cardiac | Abdominal aortic aneurysm |
|  | Arrhythmia |
|  | Congenital vascular malformation Constrictive pericarditis |
|  | Cor pulmonale |
| Renal | Chronic kidney disease |
|  | Fetal hydronephrosis |
|  | Hematuria |
|  | IgA nephropathy |
|  | Idiopathic crescentic glomerulonephritis |
|  | Kidney stones |
|  | Vesicoureteral reflux |

Supplemental Table 2. Infections before and after XLA diagnosis *

|  | Number of patients N=240 | % of patients reporting before Dx | % of patients reporting after Dx |
| --- | --- | --- | --- |
| Pneumonia | 135 (56.2) | 49 (20.4) | 88 (36.7) |
| Sinusitis | 131 (54.6) | 20 (8.3) | 111 (46.2) |
| OM | 130 (54.2) | 34 (14.2) | 101 (42.1) |
| Skin infections | 70 (29.2) | 15 (6.2) | 54 (22.5) |
| Conjunctivitis | 60 (25) | 9 (3.8) | 54 (22.5) |
| Abscess | 24 (10.0) | 10 (4.2) | 13 (5.4) |
| Opportunistic | 20 (8.3) | 8 (3.3) | 10 (4.2) |
| Bacteremia/Sepsis | 24 (10.0) | 16 (6.7) | 10 (4.2) |
| Meningitis | 18 (7.5) | 10 (4.2) | 7 (2.9) |
| Bacterial Arthritis | 14 (5.8) | 7 (2.9) | 8 (3.3) |
| Encephalitis | 14 (5.8) | 3 (1.2) | 10 (4.2) |
| Osteomyelitis | 9 (3.8) | 2 (0.8) | 1. (2.5) |

*Note that the overall column may not equal to the sum of pre-diagnosis and post-diagnosis columns due to: a) same patient may experience the same infection before and after diagnosis and b) there were patients who reported infections, but the data did not provide time information so the before or after diagnosis status was unknown

Supplemental Table 3. Respiratory manifestations reported for USIDNET XLA patients

|  | Number of individual patients reported |
| --- | --- |
| Upper respiratory |  |
| - Rhinorrhea | 10 |
| - Nasal Congestion | 7 |
| - Croup | 3 |
| Lower respiratory |  |
| - Asthma | 39 |
| - Bronchitis | 27 |
| - COPD | 26 |
| - Cough/dry cough | 23 |
| - Bronchiectasis | 22 |
| - BOOP | 2 |
| - Dyspnea | 4 |
| - Bronchiolitis | 1 |
| - Diffuse Alveolar hemorrhage | 1 |

Supplemental Table 4. Gastrointestinal manifestations reported for USIDNET XLA patients

|  | Number of individual patients reported |
| --- | --- |
| Diarrhea | 30 |
| Infectious diarrhea | 33 |
| Gastroenteritis | 13 |
| Chronic diarrhea | 22 |
| Abdominal pain | 20 |
| GERD | 18 |
| Failure to thrive | 7 |
| Failure to gain weight or underweight | 9 |
| Esophagitis/erosive esophagitis | 2 |
| Esophageal varices | 1 |
| Constipation | 9 |
| IBD or Crohn disease | 5 |
| Colitis | 8 |
| Ulcerative Colitis | 1 |
| Appendicitis | 4 |
| Cirrhosis | 2 |
| PLE | 2 |
| Celiac | 2 |
| Obesity | 2 |
| GI obstruction | 2 |
| Hematochezia | 2 |
| Diverticulitis | 1 |
| GI-Fistula | 1 |

Supplemental Table 5. Neurologic manifestations reported for USIDNET XLA patients

|  | Number of individual patients reported |
| --- | --- |
| Cognitive disability | 15 |
| Seizures | 11 |
| Speech delay | 10 |
| Milestone delay | 10 |
| Educational problems/developmental academic disorder | 9 |
| Mental retardation | 8 |
| Polio | 5 |
| Headache | 4 |
| Epilepsy | 2 |
| Dementia | 2 |
| Deafness-Dystonia-Optic Neuropathy | 2 |
| Developmental Disorder of motor function | 1 |
| Progressive Multifocal leukoencephalopathy | 1 |
| Cerebral palsy | 1 |
| Hemiplegic migraines | 1 |

Supplemental Table 6. Muscular skeletal manifestations reported for USIDNET XLA patients

|  | Number of individual patients reported |
| --- | --- |
| Arthritis | 37 |
| Arthralgias | 18 |
| Generalized aches and pain | 15 |
| Scoliosis | 5 |
| Joint swelling or stiffness | 5 |
| Dermatomyositis | 5 |
| Rheumatoid Arthritis | 3 |
| Osteoarthritis | 3 |
| Clubbing | 1 |
| Congenital Clubfoot | 1 |
| Enthesitis | 1 |

Supplemental Table 7. Hematologic manifestations reported for USIDNET XLA patients

|  | Number of individual patients reported |
| --- | --- |
| Neutropenia/congenital/autoimmune | 22 |
| Anemia / iron deficiency | 22 |
| Thrombocytopenia | 7 |
| Petechiae | 4 |
| Van Willebrand | 1 |
| HLH | 1 |
| B cell lymphoma | 1 |
| Disseminated Intravascular Coagulation | 1 |

Supplemental Table 8. Endocrine manifestations reported for USIDNET XLA patients

|  | Number of individual patients reported |
| --- | --- |
| Short Stature | 8 |
| Diabetes | 3 |
| Hypothyroidism | 2 |
| Growth hormone disorder | 2 |
| Autoimmune thyroiditis | 2 |
| Gynecomastia | 2 |
| Hyperthyroidism | 1 |
| Hypopituitarism | 1 |
| Delayed sexual development/puberty | 1 |
| Hypogonadism | 1 |
| Delayed growth | 1 |
| Goiter | 1 |

Supplemental Table 9. Psychiatric manifestations reported for USIDNET XLA patients

|  | Number of individual patients reported |
| --- | --- |
| ADHD | 14 |
| Depression | 7 |
| Behavior problems | 3 |
| Anxiety | 2 |
| Autism | 2 |
| Drug abuse | 2 |
| Suicidal thoughts | 1 |
| Socialized behavior disorder | 1 |

Supplemental Table 10. Cardiac manifestations reported for USIDNET XLA patients

|  | Number of individual patients reported |
| --- | --- |
| Cor pulmonale | 3 |
| Arrhythmia | 2 |
| Constrictive pericarditis | 1 |
| Abdominal Aortic Aneurysm | 1 |
| Congenital vascular malformation | 1 |

Supplementary Table 11. Immunomodulatory medications reported for USIDNET XLA patients

| Medication | Indication |
| --- | --- |
| Adalimumab | granulomatous dermatitis. |
| Adalimumab | autoimmune cytopenia |
| Adapalene | acne |
| Eltrombopag | thrombocytopenia |
| Filgrastim | neutropenia |
| Methotrexate | colitis |
| Methotrexate | arthritis |
| Methotrexate | morphea |
| Methylprednisolone | arthritis |
| Mycophenolate mofetil | membranoproliferative glomerulonephritis |
| PACLitaxel | angiosarcoma |
| prednisone | dental crowns |
| prednisone | asthma |
| prednisone | hives |
| prednisone | sinusitis |
| Rituximab | not reported |
| Solu-Medrol | croup |
| WinRho | acute ITP |

Supplemental Table 12: Comparison of demographic data between patients without or with *BTK* mutation.

|  | | | |
| --- | --- | --- | --- |
|  | Unknown BTK Variant^1^  N=215 | Known BTK Variant^1^  N=25 | p-value^2^ |
| Race |  |  | 0.11 |
| - White/Caucasian | 130 (60%) | 18 (72%) |  |
| - Black/African American | 21 (9.8%) | 2 (8.0%) |  |
| - Asian or Pacific Islander | 5 (2.3%) | 1 (4.0%) |  |
| - Other or more than one race | 5 (2.3%) | 2 (8.0%) |  |
| - Hispanic or Latino | 19 (17%) | 1 (4.2%) |  |
| Living Status |  |  |  |
| - Alive | 135 (88%) | 23 (92%) | 0.7 |
|  |  |  |  |
| *Patient Characteristics* |  |  |  |
| Family History | 97 (48%) | 15 (68%) | 0.2 |
| Age at disease onset | 0.8 (0.3,2.15) | 0.80 (0.28,1.28) | 0.6 |
| Age at diagnosis | 2.2 (0.8,5.0) | 1.1 (0.5,2.1) | 0.03 |
| Length of time with XLA diagnosis: | 10 (3,20) | 10 (7,14) | 0.5 |
| Body mass index (Mean, SD) | 22 (17,24) | 18 (16,22) | 0.09 |
|  |  |  |  |
| *Treatment modalities* |  |  |  |
| IgG Therapy (%) | 197 (92%) | 24 (96%) | 0.7 |

^1^ Median yrs (IQR); n (%)

^2^ Wilcoxon rank sum test; Fisher’s exact test

Supplemental Table 13. Prophylactic antibiotics reported for USIDNET XLA patients

| Medication | N=58 patients (%)  Reported 105 different antibiotics |
| --- | --- |
| Trimethoprim/Sulfamethoxazole | 26 (24.8) |
| Amoxicillin/Clavulanate | 16 (15.2) |
| Azithromycin | 13 (12.4) |
| Amoxicillin | 11(10.5) |
| Doxycycline | 8 (7.6) |
| Levofloxacin | 5 (4.8) |
| Ciprofloxacin | 4 (3.8) |
| Meropenem | 4 (3.8) |
| Cefuroxime | 3 (2.9) |
| Tinidazole | 3 (2.9) |
| Ceftriaxone | 2 (1.9) |
| Metronidazole | 2 (1.9) |
| Cefalexin | 1 (1.0) |
| Gentamicin | 1 (1.0) |
| Amphotericin | 1 (1.0) |
| Moxifloxacin | 1 (1.0) |
| Nitazoxanide | 1 (1.0) |
| Valacyclovir | 1 (1.0) |
| Voriconazole | 1 (1.0) |

Supplemental Table 14: White vs Non-White demographic differences

|  | White  N= 148^1^ | Non-White  N=36^1^ | p- value^2^ |
| --- | --- | --- | --- |
| Age of Death | 22 (17, 28) | 30 (30, 30) | 0.4 |
| Living | 96 (90%) | 31 (97%) | 0.3 |
| Age at Disease Onset (years) | 0.80 (0.30, 3.00) | 0.80 (0.50, 1.43) | 0.7 |
| Age at Diagnosis  (years) | 2.0 (0.8, 4.3) | 1.9 (0.8, 4.2) | 0.8 |
| Years since Diagnosis | 9 (3, 19) | 10 (3, 20) | >0.9 |
| BMI | 21 (17, 26) | 20 (17, 23) | 0.7 |
| Age at start of IgGR | 2.0 (0.8, 5.0) | 1.5 (1.0, 3.5) | >0.9 |
| On IgGR at last visit | 126 (100%) | 31 (97%) | 0.2 |
| Family Hx of PID | 72 (50%) | 18 (53%) |  |

^1^ Median (IQR); n (%)

^2^ Wilcoxon rank sum exact test; Wilcoxon rank test; Fisher’s exact test; {Pearson’s Chi-squared test
